# Analysis of a Chinese Pedigree With Familial Chylomicronemia Syndrome Reveals Two Novel *LPL* Mutations by Whole-Exome Sequencing

**DOI:** 10.3389/fgene.2020.00741

**Published:** 2020-07-17

**Authors:** Ying Liu, Zhangzhang Lan, Fang Zhao, Shuangchuan Zhang, Wenyong Zhang

**Affiliations:** ^1^Department of Pediatrics, Peking University Shenzhen Hospital, Shenzhen, China; ^2^School of Medicine, Southern University of Science and Technology, Shenzhen, China

**Keywords:** Familial chylomicronemia syndrome, *LPL*, whole-exome sequencing, hypertriglyceridemia, missense mutation

## Abstract

Familial chylomicronemia syndrome (FCS) is a rare monogenic autosomal recessive disease caused by loss-of-function mutations in genes involved in chylomicron breakdown through hydrolysis of triglycerides into free fatty acids. Patients are often diagnosed in early childhood with extremely high triglyceride levels and symptoms including abdominal pain, eruptive cutaneous xanthomata, hepatosplenomegaly, and significant cognitive, psychological, and social impairment. The most serious medical condition suffered by FCS patients is recurrent acute pancreatitis. Lipoprotein lipase (LPL) gene mutation accounts for majority of the known pathogenic mutations. Early diagnosis and strict low-fat diet are critical for successful management of the triglyceride concentration to lower the risk of pancreatitis. The true prevalence of FCS in China is unknown and here we report a Chinese female preterm neonate presented with an extremely high triglyceride level of 22.11 mmol/L on day 13 after birth. Clinical and laboratory workup including whole-exome sequencing revealed two novel compound heterozygous *LPL* mutations (c.406G > C and c.829G > C) that are co-segregated with her non-consanguineous parents, consistent with autosomal recessive inheritance. A diagnosis of FCS based on clinical, biochemical, and genetic ground was made to guide her management.

## Introduction

Familial chylomicronemia syndrome (FCS, OMIM 238600) is characterized by very severe hypertriglyceridemia (Berglund et al., 2012; Hegele et al., 2014). Multiple symptoms may present starting from an early age, including episodic abdominal pain, eruptive cutaneous xanthomata, and hepatosplenomegaly (Feoli-Fonseca et al., 1998). The most severe and life-threatening complication of FCS is acute pancreatitis, which is shown to have a mortality rate of 5–30% in multiple studies (Guo et al., 2014; Gaudet et al., 2016). FCS shows a classic autosomal recessive inheritance pattern, and the prevalence in the United States population is approximately 1 in 1,000,000 (Hegele et al., 2014). FCS patients are deficient in the clearance of chylomicrons due to homozygous or compound heterozygous loss-of-function mutations in genes that are involved in the catabolism of triglyceride (TG)-rich lipoproteins, including Lipoprotein lipase (*LPL*), *APOC2*, *GPIHBP1*, *APOA5*, and *LMF1*. *LPL* mutations account for 95% of all monogenic mutations associated with FCS (Murthy et al., 1996; Brahm and Hegele, 2015). Diagnosis of FCS is a complex process that involves clinical, biochemical, and genetic analysis (Beil et al., 1982). FCS may be underdiagnosed, and its true prevalence is underreported as specialized tests including *LPL* activity analysis and comprehensive genetic test may not be available when diagnosis is made (Davidson et al., 2017; Falko, 2018).

Herein, we report a Chinese female neonate with severe hypertriglyceridemia of 22.11 mmol/L. After clinical and laboratory workup, a genetic etiology was suspected and genetic testing was implemented with parents’ consent.

## Case Description

The Chinese baby girl was born at 29 + 1 week by cesarean section weighing 1300 *g* to her non-consanguineous parents. She is the first child to a 25-year-old, G2P1 mother diagnosed with systemic lupus erythematosus (SLE) during her pregnancy. The family history was otherwise unremarkable. The parents’ lipid profiles were largely within the normal range except for a slight increase of triglyceride for her mother (3.22 mmol/L). Apgar score was 9–10 at 1 min. After birth, she suffered from pneumonia and neonatal respiratory distress syndrome with symptoms including tachypnea, nasal flare, and grunting. She was admitted to NICU and received antibiotic treatment, symptomatic management, and total parenteral nutrition (TPN). She was transitioned to full gastrointestinal feeding on day 15 after birth. On day 13, her blood drawn for routine tests showed signs of chylomicronemia, and serum lipid profile was obtained and revealed severe hypertriglyceridemia (Table 1 and Supplementary Figure S1A). Blood glucose level and thyroid, liver, and kidney function tests were normal. Studies of amino acids and carnitine metabolism were unremarkable. Her markedly elevated triglyceride level was suspected to have a genetic etiology and whole-exome sequencing (WES) was performed after genetic counseling and obtaining informed written consent from the parents. This study was approved by the Scientific Research Ethics Committee of Peking University Shenzhen Hospital [(2019) NO.058].

**TABLE 1:** Patient’s lipid profile on various diet.

**Age (days)**	**Diet**	**TG**	**TC**	**HDL**	**LDL**
		
		**(mmol/L)**			
13	Preterm Formula Milk	22.11	3.20	0.25	1.76
	(fat: 4.1 *g*/100 ml, MCT: 40%)				
16	Formula Milk	15.25	3.90	0.28	2.03
21	(fat: 3.4 *g*/100 ml)	21.00	3.75	0.25	1.85
25		9.40	2.80	0.31	1.47
33		10.83	2.99	0.30	1.55
36		13.57	2.06	0.19	0.97
40		13.13	2.15	0.21	0.99
49		14.03	2.13	0.20	0.93
67	Monogen Formula Milk (MCT: 84%)	2.86	/	/	/
90	Monogen Formula Milk: (MCT: 50%)	10.35	3.73	0.38	2.15
210	Monogen Formula Milk: (MCT: 50%)	7.38	6.86	2.26	0.29

*TG, triglyceride (normal range: 0.20–2.31 mmol/L); TC, total cholesterol (normal range: 3.36–6.64 mmol/L); HDL, high-density lipoprotein (normal range: 0.83–1.96 mmol/L); LDL, low-density lipoprotein (2.07–3.1 mmol/L); and MCT, medium-chain triglyceride.*

## Methods

### Whole-Exome Sequencing and Variant Analysis

Genomic DNA was used for WES with SureSelect Human All Exon V6 array on the Illumina HiSeq X Ten platform with PE150 strategy according to the manufacturer’s instructions (Chen et al., 2015). Sequences were aligned to the human reference genome UCSC/hg19 (Kent et al., 2002), and variants were called using the GATK Unified Genotyper with the default setting and annotated using a custom annotator (McKenna et al., 2010; DePristo et al., 2011; Auwera et al., 2013). Variants with minor allele frequency >1% were removed. Variants were then filtered into four categories: *de novo*, homozygous, and two parental compound heterozygous variants. Variants were further filtered according to GeneReviews (NCBI). *In silico* analysis of variants was performed with open access software, such as SIFT^1^ and PolyPhen2^2^. Variant interpretation and prioritization were based on the clinical relevance of the gene and the pathogenicity of the variants using ACMG-AMP guidelines (Richards et al., 2015). The suspected variants were confirmed by Sanger sequencing for blood samples obtained from the patient as well as her parents.

## Results

### Genetic Analysis by WES Revealed Two Novel Compound Heterozygous Mutations in *LPL* Gene

**FIGURE 1:**
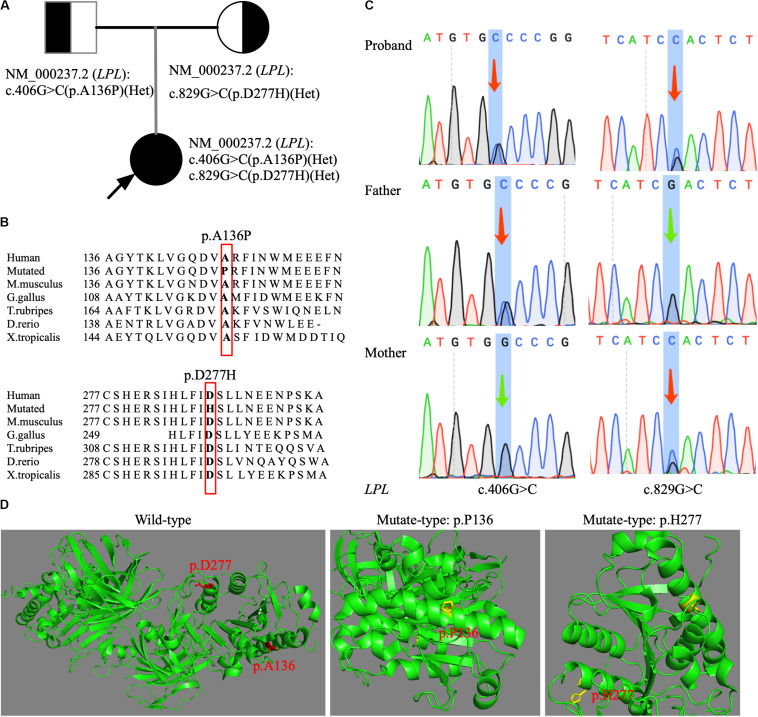
Genetic analysis of a family with FCS. **(A)** The pedigree illustrated the affected and carrier status in this family. Arrow: proband. **(B)** Sequence conservation analysis of two newly discovered *LPL* mutations (p.A136P and p.D277H) across six species by MutationTaster2. **(C)** Sanger sequencing confirmation of heterozygous status of *LPL* mutations (c.406G > C and c.829G > C) in DNA from the parents. Arrow: changed base. **(D)** Structural modeling of protein folding caused by two novel *LPL* missense mutations. The wild-type and the mutated regions are shown in red and yellow, respectively.

Variants on genes known to cause severe hypertriglyceridemia such as *LPL*, *APOC2*, *GPIHBP1*, *APOA5*, and *LMF1* were first selected and interpreted. Bioinformatic analysis revealed compound heterozygous missense mutations (c.406G > C p.A136P and c.829G > C p.D277H) in *LPL* gene. Both variants were neither found in Exome Aggregation Consortium (exac.broadinstitute.org) nor in 1000G^3^. A c.829G > A (p.A277N) transition was previously reported as a pathogenic mutation, causing a catalytically defective *LPL* protein (Ma et al., 1992). We performed sequence conservation analysis across different species, and results showed that both newly found missense mutations were highly conserved across species (*Mus musculus*, *Gallus gallus*, *Takifugu rubripes*, *Danio rerio*, and *Xenopus tropicalis*; Figure 1B). Molecular modeling of *LPL* protein with and without two-novel missense mutations using PyMOL software is shown in Figure 1D and suggested that both mutations would cause the protein to fold improperly. Furthermore, the two mutations were predicted to be deleterious by SIFT and “probably damaging” by PolyPhen, respectively. Sanger sequencing of the parents’ DNA showed that they were heterozygous carriers, with the father being a heterozygous carrier of c.406G > C (p.A136P) mutation and the mother being a heterozygous carrier of c.829G > C (p.D277H) mutation (Figures 1A,C). Using the ACMG criteria for assessing pathogenicity, p.A136P and p.D277H were ranked as likely pathogenic.

### Patient Management and Follow-Up

While in the hospital, the patient was switched from the preterm formula milk to term formula milk to lower her fat intake starting on day 15. Blood TG level decreased from 22.11 mmol/L on day 13 to 14.03 mmol/L on day 49 when she was discharged a day later. After discharge, the patient was advised to be fed on special Monogen formula milk with high medium-chain glycerides (MCT) content and to be followed up by clinicians periodically (Table 1 and Supplementary Figure S1).

## Discussion

Chylomicronemia syndrome consists of two distinctive forms: rare monogenic early onset chylomicronemia and more commonly encountered polygenic chylomicronemia of adulthood (Berglund et al., 2012; Hegele et al., 2014). The nomenclatures “Familial *LPL* deficiency,” “Type 1 hyperlipoproteinemia,” or “Familial chylomicronemia syndrome (FCS)” all describe the monogenic form that shows an autosomal recessive inheritance pattern. FCS patients are deficient in chylomicrons clearance, thus suffering from a plethora of symptoms resulting from an extremely high plasma triglyceride level.

Early diagnosis of FCS is extremely important for proper implementation of management strategies that are individually targeted to reduce potential severe consequences of extremely high blood triglycerides, particularly acute, recurrent pancreatitis. Studies have shown that there is a close correlation between acute pancreatitis risk and plasma triglyceride levels, with 3–4% increase of acute pancreatitis risk with every 100 mg/dl increase of TG concentration (Murphy et al., 2013; Rashid et al., 2016). As FCS patients often present in early childhood, a delicate balance between ensuring developmental normalcy supported by sufficiency nutrition and reducing dietary fat intake is necessary for a successful long-term management strategy. FCS is frequently misdiagnosed and patients reported seeing on average at least five physicians before a correct diagnosis is made (Gelrud et al., 2017; Falko, 2018). Such diagnostic odyssey is common in rare disease diagnosis and poses significant psychological, lifestyle, and financial stress to patients and their family members.

Our case is unusual in that the patient was a 29-week preterm neonate with multiple medical complications and presented with extremely severe hypertriglyceridemia. After ruling out other possible causes of TG elevation, a suspicion of genetic etiology led to the decision to perform a comprehensive genetic analysis using WES, whose utility has been demonstrated in rare disease diagnosis (Niguidula et al., 2018). WES is an effective diagnostic tool in detecting point mutations, indels, and small copy number variations in the coding regions. However, WES cannot reliably detect mutations in the non-coding regions, low-level mosaicism, aneuploidy, trinucleotide repeat expansions, or chromosomal structural rearrangements. Studies have shown that WES has a diagnostic yield of around 25–30% in previously undiagnosed rare disease cases (Yang et al., 2013; Fitzgerald et al., 2015). We used WES and subsequent pedigree study to successfully identify two novel compound heterozygous *LPL* gene mutations, c.406G > C p.A136P and c.829G > C p.D277H, that were each passed down to the patient from her parents. To our knowledge, both mutations were previously unreported in the public database. They most likely result in a reduction in *LPL* catalytic activity rather than affecting protein synthesis and secretion by structural analysis (Ramasamy, 2016). So far, there has been no epidemiology study of the incidence and prevalence of FCS in China. Our report suggests that FCS can be readily diagnosed in the neonates with proper workup and accurate early diagnosis will be instrumental in setting a lipid-lowering goal to prevent serious complications such as recurrent acute pancreatitis and developmental issues.

## Data Availability Statement

The datasets for this article are not publicly available due to concerns regarding participant/patient anonymity. Requests to access the datasets should be directed to the corresponding author.

## Ethics Statement

The studies involving human participants were reviewed and approved by the Scientific Research Ethics Committee of Peking University Shenzhen Hospital [(2019) NO.058]. Written informed consent to participate in this study was provided by the participants’ legal guardian/next of kin. Written informed consent was obtained from the patient’ parents for the publication of any potentially identifiable images or data included in this article.

## Author Contributions

YL contributed to the data collection, data interpretation, and genetic counseling. ZL contributed to the study design and writing of the manuscript. FZ and SZ contributed to the clinical data collection and data interpretation. WZ conceived of the study, participated in its design and coordination and revising the article critically for important intellectual content. All authors contributed to the article and approved the submitted version.

## Conflict of Interest

The authors declare that the research was conducted in the absence of any commercial or financial relationships that could be construed as a potential conflict of interest.

## Footnotes

1 https://sift.bii.a-star.edu.sg/

2 http://genetics.bwh.harvard.edu/pph2/index.shtml

3 https://www.internationalgenome.org/1000-genomes-browsers
