# Supplementary figures and images for: Analysis of a Chinese Pedigree With Familial Chylomicronemia Syndrome Reveals Two Novel LPL Mutations by Whole-Exome Sequencing
Source: Front Genet. 2020 Jul 17;11:741. doi: 10.3389/fgene.2020.00741 (PMC7379882; doi:10.3389/fgene.2020.00741)

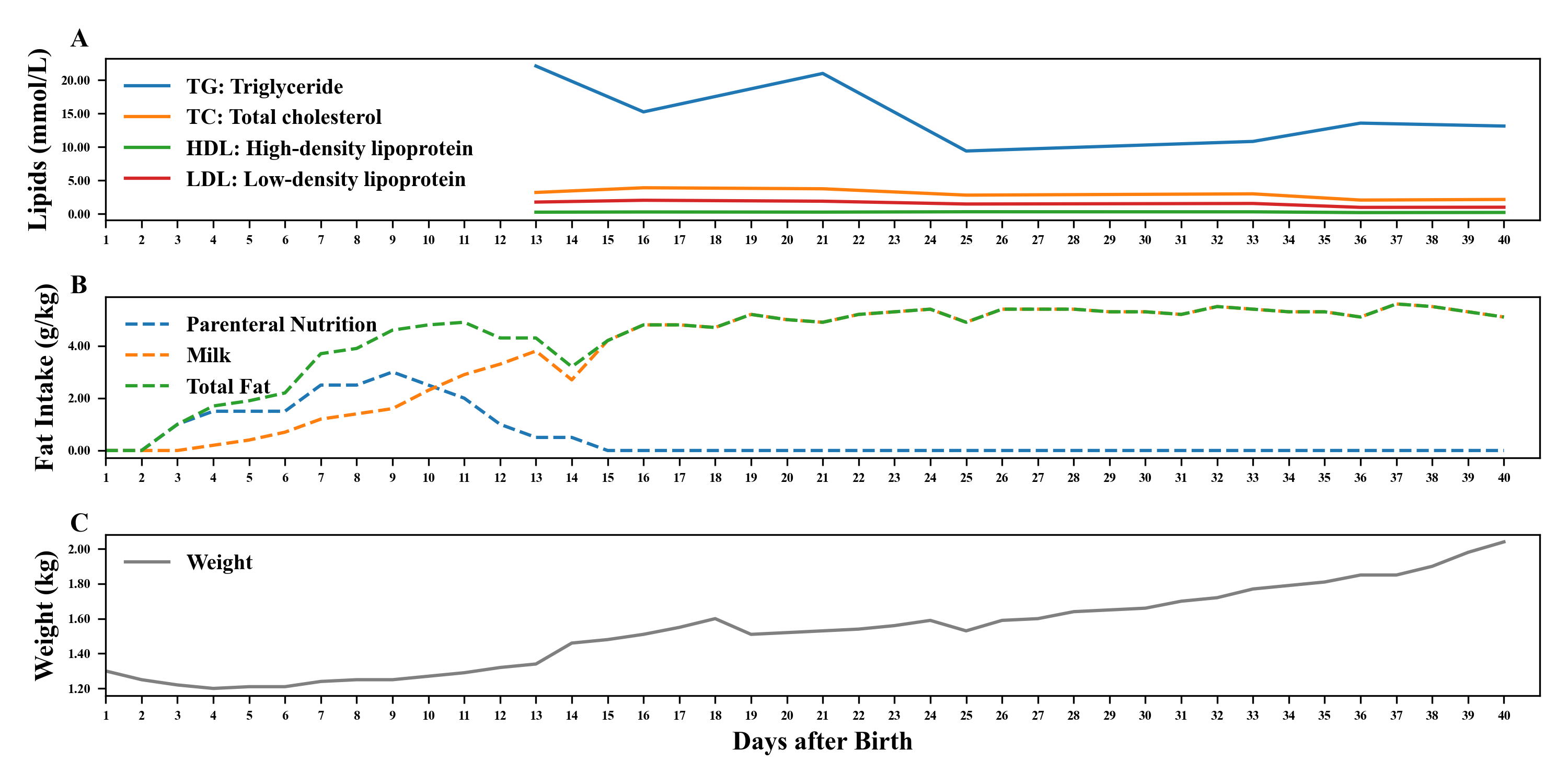

Supplement: Supplementary file 1 [file Image_1.TIFF]
